# Bladder hematoma: a complication from an oocyte retrieval procedure

**DOI:** 10.5935/1518-0557.20180086

**Published:** 2019

**Authors:** Maria do Carmo Borges de Souza, Marcelo Marinho de Souza, Roberto de Azevedo Antunes, Maria Augusta Tamm, Joyce Barreto da Silva, Ana Cristina Allemand Mancebo

**Affiliations:** 1 Fertipraxis-Centro de Reprodução Humana- RJ, Brazil

**Keywords:** Hematoma, urinary tract, *in vitro* fertilization, complications

## Abstract

**Introduction:**

More than one million fertilization cycles are performed every year. The incidence of serious complications associated with transvaginal oocyte pick-up is low, but the procedure is not risk-free. Risks are inherent to procedures in which thin needles and sharp instruments are introduced into the vaginal wall and ovarian capsule to access the ovaries.

**Case Description:**

A 45-year-old patient reported urinary discomfort and difficulty urinating after her second cycle, 12 hours after oocyte pick-up. She had visible hematuria with small blood clots. Transvaginal ultrasound examination performed 24 hours after pick-up showed a heterogeneous intravesical image suggestive of a clot; her bladder measured 23x19mm. She was afebrile and in good condition. The patient was managed conservatively and offered fluids. The clot was expelled within a matter of hours. This case of a bladder hematoma was the first in the 21 years of a clinic where all procedures are guided by ultrasonography with clear visualization of the tip of the needle throughout the 15-20 minutes of the procedure. Patients submitted to ultrasound-guided transvaginal oocyte pick-up procedures in IVF protocols must be informed of this rare potential complication.

## INTRODUCTION

Transvaginal ultrasound-guided follicular aspiration was first described in 1983 and rapidly became widely accepted because of its simplicity and effectiveness (Gleicher *et al*., 1983; Bennett *et al*., 1993). In 2013, the International Committee Monitoring Assisted Reproductive Technologies (ICMART) estimated that 6.5 million children were born in the world from *in vitro* fertilization (IVF) procedures and that more than one million annual follicular punctures were performed in at least 60 countries (Adamson *et al.*, 2017).

According to the literature, the incidence of serious complications associated with transvaginal oocyte pick-up is low, but the procedure is not risk-free. Risks are inherent to procedures in which thin needles and sharp instruments are introduced into the vaginal wall and ovarian capsule to access the ovaries. A transvaginal probe equipped with a needle guide is introduced into the vaginal canal and positioned in the lateral vaginal fornix on the same side of the ovary to be aspirated. This is done in order to reach the gonad with the end of the probe as closely as possible (Seyhan *et al*., 2014). The needle is then connected to a suction pump, and introduced into the follicles after perforation of the vaginal wall and ovarian capsule. Only then the aspiration of the follicular fluid begins.

As a precaution, the number of vaginal and ovarian perforations is kept to a minimum. Therefore, greater numbers of follicles are aspirated without withdrawing the needle tip from inside the ovary (el Hussein *et al*., 1992). After all ovarian follicles have been aspirated, the needle is withdrawn and the procedure is repeated in the contralateral ovary. The tip of the needle must be visualized by ultrasound throughout the entire procedure in order to avoid damaging adjacent pelvic structures (Gleicher *et al*., 1983).

## CASE REPORT

The patient described in this paper consented to having her case published. A single nulliparous woman was first seen in our clinic in April 2014. She was seeking information on oocyte cryopreservation and *in vitro* fertilization. She did not have a partner at the time. She had no record of comorbidities and her menstrual cycles were regular. The antimullerian hormone (AMH) level measured after her first appointment was 3.4 ng/mL and her antral follicle count (AFC) was 12. She returned to the clinic in September 2015 with a partner (age 38). At the beginning of the cycle, her FSH dosage was 6 mIU/mL; her estradiol (E2) level was 43 ng/dL; and her AFC was 17. Her prospects in relation to age were discussed and she was offered an IVF/ICSI cycle with genetic testing for aneuploidies of the resulting blastocysts.

The protocol and total amount of gonadotropins administered were as follows: GonalF^®^ (recombinant FSH, Merck, Aubonne, Switzerland) 1050 IU and Menopur^®^ (Ferring Pharmaceuticals, Kiel, Germany) 1050 IU. This patient was prescribed an antagonist cycle with Orgalutran^®^ (Merck Sharp & Dohme, Ravensburg, Germany), 4 vials. The LH trigger utilized was Ovidrel^®^ 250 mcg (recombinant HCG, Merck, Aubonne, Switzerland). The transvaginal ultrasound-guided oocyte retrieval procedure was performed in September 2015 with a standard Wallace^®^ (UK) 25-cm 17-G single-lumen needle attached to a closed suction system with a continuous pressure pump at 90 mmHg, as per the protocol in place in our Center. Ten oocytes were harvested, 4 of which were Metaphase II; two embryos were vitrified on day 3.

The patient underwent a second cycle in April 2016, at age 45, with Pergoveris^®^ (recombinant FSH plus recombinant LH, Merck, Aubonne, Switzerland), using a total gonadotropin dose of 1350 IU of FSH and 670 IU of LH, and an additional 1200 IU of FSH with Fostimon M^®^ (IBSA Institut Biochimique S.A. Lamone, Switzerland). The patient was again prescribed an antagonist cycle with Orgalutran^®^ (Merck Sharp & Dohme, Ravensburg, Germany), 5 vials. This time the LH trigger was performed with Gonapeptyl daily^®^ 0.1mg (Ferring Pharmaceuticals, Kiel, Germany), 2 vials. However, during the second cycle she decided to only cryopreserve her oocytes. All pre-procedure examinations were normal. Follicle aspiration was done 35 hours after the LH trigger. Both ovaries were punctured without complications. A Kitazato^®^, Japan, 17-G needle was used in the procedure and nine metaphase II oocytes were harvested. Immediately prior to aspiration, the patient was instructed to void her bladder to reduce the contact area with the puncture zone. An experienced team carried out the aspiration procedure and no issues were detected.

**Figure 1:**
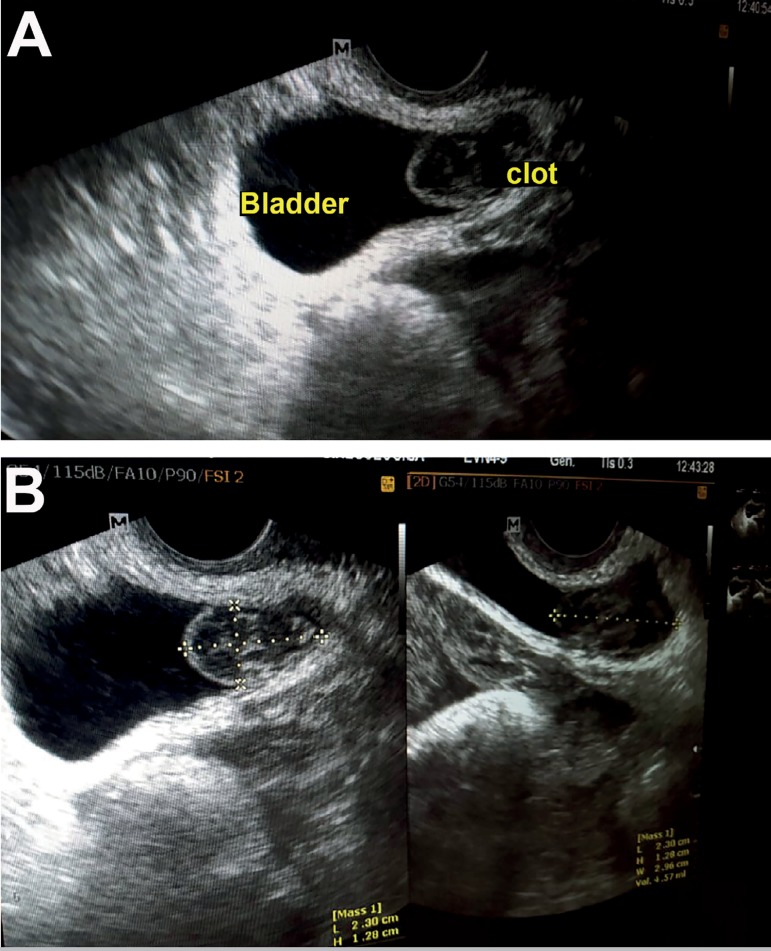
A and B. Transvaginal ultrasound examination performed one day after oocyte retrieval, showing a heterogeneous intravesical image and a well-defined bladder measuring 23x19mm in its larger diameter

The patient reported urinary discomfort and difficulty voiding her bladder 12 hours after follicular puncture. She reported seeing blood and small blood clots in her urine. However, she only contacted the clinic the following day. She was asked to return immediately to the clinic. Despite the complaints, the patient was in good general condition and without signs of fever within 24 hours of the pick-up procedure. Transvaginal ultrasound examination performed 24 hours after pick-up showed a heterogeneous intravesical image (Figure 1) suggestive of a clot; her bladder measured 23x19mm. The urologist with the team recommended an expectant approach with increased fluid intake. The patient reported discomfort throughout the day. She expelled the clot in the evening, and symptoms subsided. She has not recurred in the months following this episode.

## DISCUSSION

This was the only case of a bladder hematoma in more than 2739 procedures (0.03%), which now appear alongside one case of a hematoma of the parametrium and another case of hemoperitoneum. All oocyte pick-up procedures were ultrasound guided. Additionally, clear visualization of the needle tip is mandatory throughout the procedure.

The patient described in this case had no history of pelvic or abdominal surgery. Although the rate of complications associated with vaginal oocyte pick-up is low, care and attention are mandatory in order to minimize potential harm. Patients must be checked for prior pelvic surgery, sequelae from pelvic inflammatory disease, and history of endometriosis.

According to Bennett *et al*. (1993), vascular lesions of the vaginal and ovarian walls, accidental injuries to pelvic organs such as the bowel, bladder, ureters and pelvic blood vessels, and pelvic infection by microorganisms from the vaginal canal are a few of the possible complications arising from this procedure. These authors reviewed 2670 cases of oocyte retrieval and described vaginal bleeding (8.6%) as the most frequent complication, followed by hemoperitoneum (0.7%), pelvic infection (0.6%), and accidental puncture of pelvic vessels (0.04%).

Ludwig *et al.* (2006) described similar findings. The authors examined the peri- and postoperative complications of 1058 oocyte retrieval procedures and found vaginal bleeding (2-3%) as the most frequent complication, followed by hemoperitoneum (1 %). They did not report cases of pelvic infection, although it appears to occur in 0.2 to 0.6% of the cases (Dicker *et al*., 1993).

Seyhan *et al*. (2014) reported similar findings in a comparison between complication rates and pain score definitions after oocyte retrieval for *in vitro* maturation and IVF cycles. Vaginal and ovarian bleedings were the most frequent complications. Their findings were in agreement with previous studies, in which vaginal bleeding occurred in 0.5-7.5% of the cases and pelvic pain was the most frequent complication (el Hussein *et al.*, 1992; El-Shawarby *et al*., 2004). El-Shawarby *et al*. (2004) also described other complications including adnexal torsion, ruptured endometriotic cysts, issues with anesthesia, and vertebral osteomyelitis.

The literature has been unanimous in showing that complications arising from oocyte retrieval are rare. In this context, accidental urinary tract injuries are apparently even less frequent. von Eye Corleta *et al.* (2008) reported a case of immediate ureterovaginal fistula secondary to oocyte retrieval, which improved spontaneously after six weeks. Similarly, Jones *et al*. (1989) reported three other cases of ureteral injury. Coroleu *et al*. (1997) and Fugita & Kavoussi (2001) described cases in which patients were diagnosed with complications between five days and four months after the retrieval procedure, involving a combination of irritative voiding symptoms, leukocytosis, and negative urine culture, which, according to them, indicated urinary tract injury. These authors also emphasized the importance of early diagnosis. In fact, Miller *et al*. (2002) reported a case of acute ureteral obstruction following a seemingly uncomplicated oocyte retrieval procedure, in which prompt diagnosis and ureteral stenting led to rapid recovery with no long-term urinary tract sequelae.

One might assume that bladder injury occurs more frequently than ureter lesions on account of the local anatomy, although this idea has not been supported by literature reports. The topographic characteristics of the bladder and its direct relationship with the site of puncture might increase the risk of injury when the needle is inserted, while the pressure exerted by the probe causes its walls to collapse, thus making visualization more difficult.

Preventing damage to pelvic structures during oocyte retrieval includes using Color-Doppler velocimetry to identify blood vessels in cases of doubt (Bhandari *et al*., 2015). Additionally, it is wise to keep the end of the needle guide always in a lateral position before puncturing to avoid being too close to blood vessels, the bladder, and the ureter. Finally, oocyte pick-up should only commence after complete bladder voiding (Miller *et al*., 2002).

As pointed out by von Eye Corleta *et al*. (2008), given the elective nature of transvaginal ultrasound-guided oocyte retrieval in IVF cycles, patients should be informed about these potential, albeit rare, risks and complications.
